# Applications of pyroptosis activators in tumor immunotherapy

**DOI:** 10.1016/j.mtbio.2024.101191

**Published:** 2024-08-06

**Authors:** Xin Bao, Mengmeng Sun, Lingfei Meng, Hong Zhang, Xuan Yi, Peng Zhang

**Affiliations:** a Department of Thyroid, The Second Hospital of Jilin University, Changchun, 130061, PR China; b Key Laboratory of Polymer Ecomaterials, Jilin Biomedical Polymers Engineering Laboratory, Changchun Institute of Applied Chemistry, Chinese Academy of Sciences, Changchun, 130022, PR China; c Department of Nephrology, The Second Hospital of Jilin University, Changchun, 130061, PR China

**Keywords:** Gasdermin, Immunotherapy, Immunogenic cell death, Pyroptosis, Tumor microenvironment

## Abstract

Contemporary progress in tumor immunotherapy has solidified its role as an effective approach in combating cancer. Nonetheless, the prevalent “immune cold” state within the tumor microenvironment poses a substantial barrier to its efficacy. Addressing this, pyroptosis—a gasdermin-mediated programmed cell death characterized by its inflammatory profile—emerges as a crucial mechanism. It catalyzes the release of vast quantities of pro-inflammatory cytokines and immunogens, potentially transforming immunosuppressive “cold” tumors into reactive “hot” ones. Herein, we will initially present an overview of pyroptosis as a distinct form of cell death, along with its molecular mechanisms. Subsequently, we will focus on introducing how pyroptosis activators are utilized in the field of tumor immunotherapy. Insights gained from applications of pyroptosis activators in tumor immunotherapy could lead to the development of safe and efficient pyroptosis activators, significantly enriching the arsenal for tumor immunotherapy.

## Graphical abstract

**Figure:**
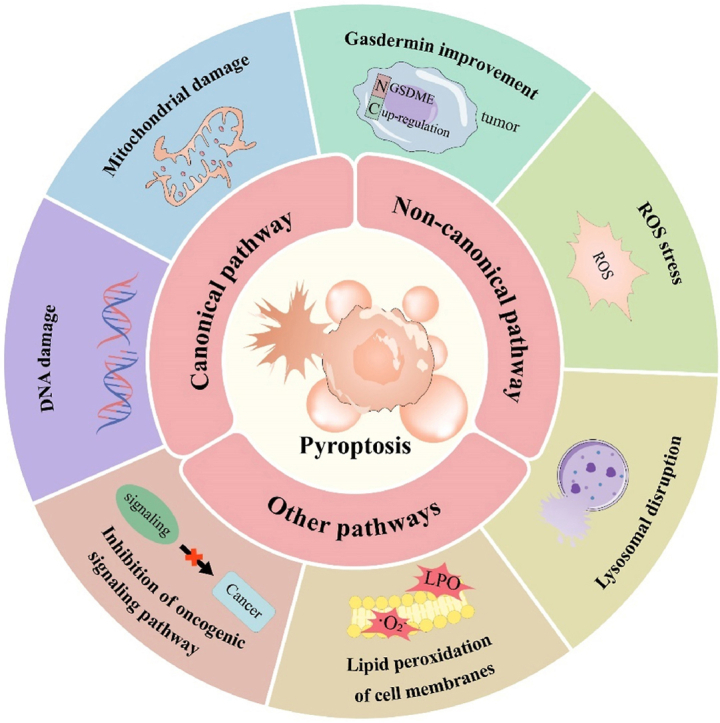

## 1 Introduction

Recent advancements in various cancer treatments including surgery, chemotherapy, radiation, phototherapy, and gene therapy have demonstrated the potentials in curbing cancer growth. However, the high rates of tumor metastasis and recurrence continue to drive significant mortality [[1], [2], [3], [4]]. Immunotherapy has emerged as a promising approach by enhancing a patient's immune capabilities to actively or passively destroy tumor cells, showing substantial progress in tumor eradication. However, the efficacy of immunotherapy largely hinges on the immunogenicity of tumors, the highly immunogenic ("hot") tumors respond better to this treatment modality. In contrast, most human tumors are "cold" with low immunogenicity, making immunotherapy effective in only a limited array of cancer types [5]. Besides, the restricted success of immunotherapy in treating most solid tumors stems from the tumor microenvironment's suppressive components, such as M2 macrophages, T regulatory cells, myeloid-derived suppressor cells (MDSCs), and inhibitory receptors including programmed cell death protein 1 (PD-1), cytotoxic T lymphocyte-associated protein-4 **(**CTLA-4), and T cell immunoglobulin mucin-receptor 3 (TIM-3), which all dampen systemic immune responses [[6], [7], [8], [9], [10], [11]]. Modern immune checkpoint blockade (ICB) therapy offers a promising direction for cancer immunotherapy [12]. The main types of ICB therapy include PD-1/PD-L1 inhibitors and CTLA-4 inhibitors, which block immune checkpoint proteins, allowing the immune system to recognize and attack tumor cells. Although ICB therapy has shown great efficacy in clinical trials, not all patients can benefit from it, and some may not respond to ICB therapy or even suffer from severe side effects [13].

As a form of cell death characterized by lysis and strong pro-inflammatory properties, pyroptosis holds significant importance for the immune system [14]. Pyroptosis can trigger cell death through distinct cellular mechanisms including pore formation on cell membranes, leading to extensive cellular swelling and the subsequent rupture of cell membranes [15,16]. This process is initiated by the activation of inflammasomes under inflammatory conditions, which in turn activate caspases that specifically cleave gasdermin (GSDM). This cleavage results in the separation of the GSDM-N and GSDM-C domains, with the GSDM-N domain migrating to the cell membrane to facilitate the formation of membrane pores. This event escalates into cell expansion, rupture, and ultimately, cell death. Concurrently, the cell rupture discharges significant quantities of inflammatory cytokines, including interleukin-1β (IL-1β) and interleukin-18 (IL-18) [17]. Groundbreaking research published in Nature in 2020 highlighted that pyroptosis is not only effective in directly eliminating tumor cells but also plays a pivotal role in provoking robust anti-tumor immune responses through the inflammatory reactions it induces [18,19]. The pyroptosis of a small number of tumor cells is sufficient to trigger an inflammatory response, improve the tumor immune microenvironment, and activate T cell-mediated antitumor immune responses [20]. Furthermore, pyroptosis is a form of immunogenic cell death (ICD) that can promote the release of tumor antigens, enhance the immunogenicity of tumors, and regulate the proportions of tumor-infiltrating immune cells such as T cells, natural killer (NK) cells, dendritic cells (DCs), monocytes, and MDSCs. This process changes the tumor immune microenvironment and transforms the "cold" tumors into the "hot" ones, thereby enhancing the therapeutic efficacy of ICB therapy [21]. Thus, pyroptosis is not only a unique form of cell death but also provides new perspectives and strategies for the immunotherapy of cancer.

**Fig. 1:**
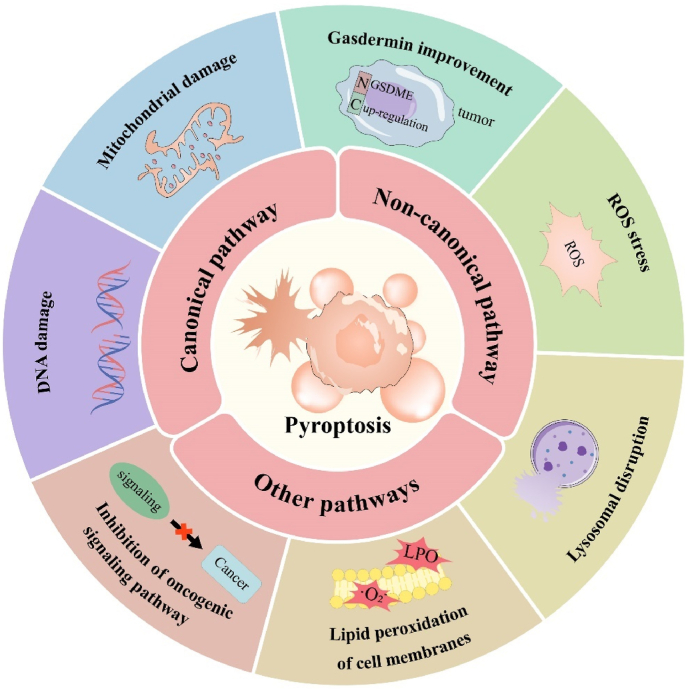
Schematic overview of applications of pyroptosis activators in tumor immunotherapy.

In this review, we will first introduce pyroptosis as a unique form of cell death, and the molecular mechanisms of pyroptosis, and then we will summarize the applications of pyroptosis activators in tumor immunotherapy (Fig. 1). By comparing the strategies of activating pyroptosis with different pyroptosis activators (Table 2), we hope to develop or re-explore more effective pyroptosis activators, bringing new hope for tumor immunotherapy.

## 2 Pyroptosis——a unique form of cell death

The forms of cell death refer to the various ways in which cellular life activities are terminated, with differences in inducers, activation pathways, morphological changes, cell death mechanism and immune effects (Table 1) [22,23]. Among them, pyroptosis stands out as a GSDM-mediated cell death triggered by diverse stimuli and has been extensively investigated in numerous diseases. Unlike other types of cell death such as apoptosis and necrosis, pyroptosis is characterized by the formation of pores in the cell membranes, leading to the release of cellular contents and subsequently triggering an inflammatory response [24]. The GSDM family, key players in pyroptosis, includes members such as gasdermin A (GSDMA), gasdermin B (GSDMB), gasdermin C (GSDMC), gasdermin D (GSDMD), gasdermin E (GSDME), and DFNB59. Except for DFNB59, these proteins consist of a C-terminal and an N-terminal domain. Upon cleavage, the N-terminal domain of GSDM is released, which can perforate the cell membranes. This action leads to distinctive morphological changes, including cytoplasmic swelling, membrane rupture, and the release of inflammatory mediators [20].

**Table 1:** Comparison of different forms of cell death.

Form of cell death	Inducers	Activation pathways	Morphological changes	Cell death mechanism	Immune effects
Apoptosis	TNF-α, FasL, TRAIL, Hypoxia, Irradiation, Heat shock, etc.	Bcl-2 protein family, P53, Caspase-2/3/6/7/8/9/10, HSPs	Plasma membrane blebbing, cellular volume reduction, nuclear fragmentation, and chromatin condensation	An orderly and programmed form of cell death involving the activation of caspase enzymes.	Most anti-inflammatory. Pro-inflammatory when DAMPs release
Necrosis	Microbial infection, Toxins, Trauma, Ischemia, Thermal stress, etc.	Unspecific	Plasma membrane rupture, organelle swelling, DNA degradation	An unordered, unprogrammed form of cell death, usually caused by external injury.	Pro-inflammatory
Pyroptosis	DAMPs, PAMPs, Microbial infection, etc.	GSDM protein family, Caspase-1/3/4/5/8/11, inflammasomes	Cells swelling, pore formation on cell membranes, cell rupture, bubbling of plasma membranes, and chromatin condensation	A form of programmed cell death mediated by the GSDM family, characterized by inflammatory features.	Pro-inflammatory
Autophagy	Hypoxia, AMPK, ULKl, etc.	AMPK, ULK, VPS34	Autophagic vacuolization	a process of cellular self-digestion involving the degradation and recycling of cellular organelles and cytoplasmic components.	Pro-inflammatory

**Table 2:** Summary of the pyroptosis activators and their activation mechanism of pyroptosis.

Pyroptosis activators	Mechanism	Immune effect	Cell type	Ref.
Pyroptosis activators that cause mitochondrial damage				
OPDEA-PDCA	I. Inhibition of mitochondrial PDHK1 II. Mitochondrial oxidative stress III. Caspase-1/GSDMD	Release HMGB1 and IL-1β	K7	[74]
CaNMs	I. Ca^2+^ overload II. Cytochrome C release III. Caspase-3/GSDME	Release inflammatory molecules and cell contents	4T1	[77]
NCyNH_2_/PEG-*b*-PLGA	I. Cytochrome C release II. Promote ROS generation II. Caspase-3/GSDME	I. Release HMGB1, IL-1β, and ATP II. Promote T cell infiltration and DC maturation	4T1, CT26	[78]
**Pyroptosis activators that cause lysosomal disruption**				
Lip-MOF	I. Lysosomal rupture II. Fe^2+^ release III. Caspase-1/GSDMD	Release LDH and IL-1β	4T1	[85]
TiO_2_@Ru@siRNA	I. Pomote ROS generation II. Lysosomal disruption III. Caspase-3/GSDME	I. Downregulation of key immunosuppressive factors II. Upregulation of immune cytokines III. Activation of CD4^+^ and CD8^+^ T cells	OSCC	[86]
ANPS	I. Activation of PLC II. Lipid peroxidation in early endosomes III. Caspase-3/GSDME	Release LDH, HMGB1, and ATP	A549, A431, MCF-7, HN5	[87]
**Pyroptosis activators that cause lipid peroxidation of cell membranes**				
TBD-3C	I. Pomote ROS generation II. Membrane damage III. Caspase-1/GSDMD	I. Release LDH, IL-1β, and IL-18 II. Stimulation of M1-polarization of macrophages III. Maturation of DCs and activation of CD8^+^ T cells	4T1, Hela, C6, Panc02	[98,103]
CA-Re	I. Promote ROS production *in situ* II. Disruption of membrane integrity II. Caspase-1/GSDMD	I. Release HMGB1, ATP, and CRT II. Promote the maturation and antigen-raising ability of DCs and activate T cells	MDA-MB-231 cells	[104]
D1	I. Pomote ROS generation II. Direct cell membrane damage III. Caspase-1/GSDMD	I. Release cell contents and inflammatory cytokines II. Promote the maturation and antigen-raising ability of DCs and activate T cells	4T1	[107]

**Pyroptosis activators that cause GSDM improvement**					
**DNAdemethylation**	BNP	I. Cytoplasm Ca^2+^ concentration increase II. Cytochrome C release III. Up-regulate GSDME expression IV. Caspase-3/GSDME	I. Release LDH II. Promote the secretion of IL-6, TNF-α, and the maturation of DCs III. Promote CD4^+^, CD8^+^ T cells infiltration and secret IFN-γ	4T1	[113]
	ANP	I. Activation of caspase-3 II. Up-regulate GSDME expression III. Caspase-3/GSDME	I. Release proinflammatory cytokines II. Promotion of CD4^+^ and CD8^+^ T cells III. Reduction of MDSCs and Treg cells	4T1	[114]
	TD@COFs	I. Activation of caspase-3 expression by ROS generation II. Up-regulate GSDME expression III. Caspase-3/GSDME	I. Stimulate T cell proliferation and DC maturation II. Infiltration of CD8^+^ T cells III. Alleviation of Treg cells	4T1	[115]
	(M + H)@ZIF/HA	I. GSDME up-regulation II. Caspase-3/GSDME	I. Promote DC maturation and T cells infiltration II. Decrease MDSCs and Treg cells III. Release TNF-α and IFN-γ	4T1	[118]
	AOZN	I. GSDMD up-regulation II. Activation of caspase-1 by increasing ATP levels II. caspase-1/GSDMD	I. Release HMGB1, and ATP II. Increase the expression of CD73, PD-L1, and IFN-γ III. Promote T cell infiltration and DC maturation	B16F10, CT26	[127]
**Others**	Phe-BF_3,_ NP-GSDMA3	Intracellular delivery of GSDMA3	I. Release IL-1β, IL-18, and HMGB1 II. Increase CD3^+^, CD4^+^, CD8^+^ T cells, NK cells, and M1 macrophage infiltration III. Decrease the presence of CD4^+^FOXP3^+^ Treg cells and M2 macrophage	HeLa, EMT6, 4T1	[18]
	Nano-CD	I. trigger endogenous GDSME expression II. Activation of caspase-3 III. Caspase-3/GSDM	Promote DC maturation, antigen presentation, T cell priming and ICD	B16F10	[128]

**Pyroptosis activators that cause DNA damage**				
PDNP	I. DNA damage caused by DOX II. Caspase-3 activation by DOX II. Caspase-3/GSDME	I. Release ATP, LDH, and HMGB1 II. facilitate DC maturation, prime T cell proliferation, and inhibit MDSCs	CT26	[133]
BIK	I. DNA damage caused by DOX II. Caspase-3 activation by ultralow-dose DOX III. Caspase-3/GSDME	I. Release high level of cytokines II. Increase CD3^+^, CD4^+^, and CD8^+^ T cells III. Reduction of Treg cells	4T1	[134]
Pt1, Pt2	I. photodamage to ncDNA II. caspase-1/GSDMD	I. Release IFN-β, TNF-α, and IL-6 II. Promote DC maturation and T cell activation	Hela	[137]
**Pyroptosis activators that cause inhibition of oncogenic signaling pathway**				
PNM	I. Inhibition of oncogenic signaling II. Caspase-3 activation III. Caspase-3/GSDME	I. Release HMGB1 and ATP II. Inhibit immunosuppressive cell populations III. Promote DC maturation and infiltration of T cells	4T1	[147]

**Pyroptosis activators that cause ROS stress**					
**Phototherapy**	MCPP	I. ROS stress by PDT II. GSDME activation by PTX III. Caspase-3/GSDME	I. Promote CRT translocation and release HMGB1 II. Induce DC maturation, T cell expansion	CT26	[158]
	CANPs	I. ROS stress by PDT II. Caspase-3/GSDME	I. Release HMGB1 and LDH II. Promote T cell infiltration and DC maturation III. Inhibit the immunosuppressive activity of MDSCs	4T1	[159]
	COF-919	I. ROS stress by PTT and PDT II. Caspase-3/GSDME	I. Release HMGB1, LDH and ATP II. Promote T cell infiltration, reduce the proportion of Tregs and MDSCs	4T1	[162]
	NI-TA	I. O_2_^−•^ generation by photocatalysis II. Caspase-3/GSDME	I. Release cell contents	T47D	[165]
**CDT**	COF-909-Cu	I. ROS stress by PTT and CDT II. Caspase-3/GSDME	I. Release HMGB1, LDH and ATP II. Decrease the population of Tregs and MDSCs III. Increase memory T cells	4T1	[171]
	Tf-LipoMof@PL	I. ROS stress by CDT II. Caspase-1/GSDMD	Release LDH and IL-1β	4T1	[175]
**Imbalance of intracellular osmolarity**	ZrNPs	I. A surge in intracellular osmolarity II. Promote ROS generation III. NLRP3/caspase-1/GSDMD	I. Release IL-1β II. Enhance DC maturity and frequency of effector-memory T cells	4T1	[185]
	PNSO NPs	I. Increase osmolarity II. ROS storm III. Caspase-1/GSDMD	I. Release IL-1β, HMGB1, ATP, and LDH II. Trigger DC maturation III. Activate CD8^+^ and CD4^+^ T cells	4T1, CT26	[186]
	NaHCO_3_ NPs	I. Regulate lactic acid metabolism II. A surge in intracellular osmolarity III. Promote ROS generation IV. caspase-1/GSDMD	I. Release DAMPs and IL-1β II. Promote the high frequency of CD8^+^ T cells, mature DCs and M1 macrophage III. Decrease the percentages of M2 macrophage, MDSCs and Treg	4T1	[187]
**ROS generators**	GOD/PICsomes	Oxidative stress	I. Release LDH, IL-1β, IL-18, and HMGB1 II. Increase CRT expression	4T1	[188]

Pyroptosis leads to the rupture of the cell membrane, releasing intracellular inflammatory factors and danger-associated molecular patterns (DAMPs), which can trigger an inflammatory response, activate the immune system, and help recruit immune cells to the tumor site, thereby enhancing immune surveillance and clearance towards the tumor. In addition, pyroptosis can change the tumor microenvironment, transforming it from an immunosuppressive state to an immunoactive state, which is conducive to the infiltration of immune cells to achieve anti-tumor effects [25]. Therefore, the inflammatory response caused by pyroptosis can work in synergy with ICB therapy to improve the efficacy of anti-tumor immune therapy [26]. In summary, pyroptosis plays an important regulatory role in tumor immunotherapy, providing strategies for tumor treatment by activating the immune system and changing the tumor microenvironment.

### 2.1 Molecular mechanisms of pyroptosis

**Fig. 2:**
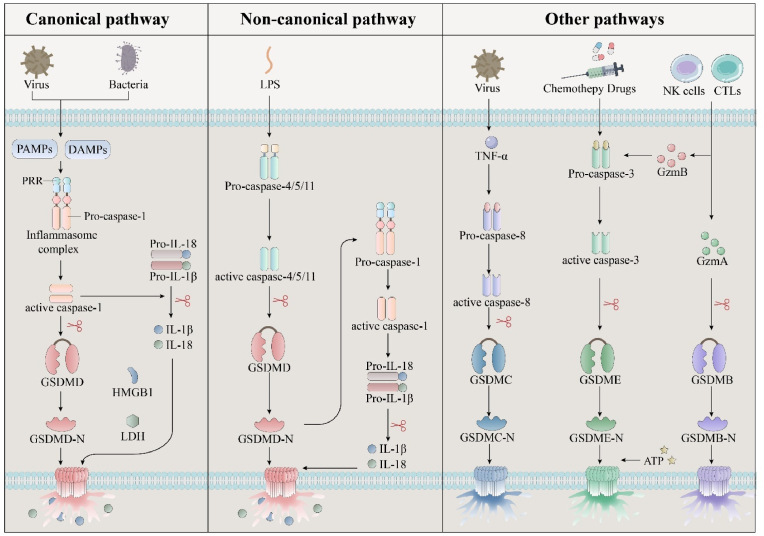
Schematic diagram of molecular mechanisms of pyroptosis.

In the innate immune response, the classical inflammasome pathway and the non-canonical inflammasome pathway can activate the GSDM family's GSDMD to mediate pyroptosis, thereby antagonizing and clearing pathogenic bacterial infections. In addition, there are other pathways, such as the activation of the GSDM family's GSDMB, GSDMC, and GSDME to mediate pyroptosis through virus, chemotherapeutic drugs, NK cells and cytotoxic T lymphocytes (CTLs) (Fig. 2).

#### 2.1.1 Canonical pathway

The classical pyroptosis pathway is primarily triggered by inflammasome activation, which is orchestrated by the inflammatorome and caspase-1. This activation sequence results in a range of pathophysiological responses that significantly influence tumor biology, including cellular proliferation, metastasis, and invasion [[27], [28], [29], [30], [31], [32], [33], [34], [35]]. Central to this pathway is the inflammasome, a complex consisting of a pattern recognition receptor (PRR), an apoptosis-associated speck-like protein (ASC), and an inactive precursor of caspase-1 [[36], [37], [38]]. Specific PRRs within this complex, such as members of the NOD-like receptor (NLR) family (NLRP1, NLRP3, NLRC4, and AIM2) and pyrin [39,40], detect pathogen-associated molecular patterns (PAMPs)—like viral dsDNA, β-glucan, and lipopolysaccharides—and DAMPs from stressed or dying cells [41]. ASC is composed of a pyrin domain (PYD) at the N-terminus and a caspase activation and recruitment domain (CARD) at the C-terminus [42,43]. Interaction between PRRs and their ligands activates a signaling cascade that culminates in inflammasome assembly, facilitated by the binding of the PYD domain of a PRR to the PYD domain of ASC and subsequent recruitment of pro-caspase-1 through CARD-CARD interactions [[44], [45], [46], [47], [48], [49], [50], [51]]. Upon activation, caspase-1 cleaves GSDMD, generating a 22 kDa C-terminal fragment (C-GSDMD) and a 31 kDa N-terminal fragment (N-GSDMD). C-GSDMD deactivates GSDMD's autoinhibition, enabling N-GSDMD to form nonselective pores about 10–14 nm in diameter in the cell membrane, leading to cellular swelling and pyroptosis [37,52,53]. Additionally, caspase-1 facilitates the maturation of IL-18 and IL-1β, which are then released through these pores, thereby playing a crucial role in inflammatory responses and cell death [54]. These findings are aligned with the pathways documented to lead to pyroptosis [55].

#### 2.1.2 Non-canonical pathway

The non-classical pathway of cell pyroptosis operates primarily through caspase-4, caspase-5, and caspase-11, functioning independently from caspase-1 [56]. In the cytoplasm, these caspases bind to and are activated by LPS from certain gram-negative bacteria, which are linked to their pathogenicity [57,58]. Upon activation, these caspases cleave GSDMD, resulting in the release of the GSDMD-C fragment and the GSDMD-N pore-forming fragment. Oligomerization relieves the intramolecular inhibition of the GSDMD-N domain due to conformational changes, facilitating the integration of the GSDMD-N terminus with the cell membrane phospholipids. This interaction leads to the creation of transmembrane β-barrel pores, causing potassium outflow, cell swelling, and rupture, which culminates in cell pyroptosis [57,[59], [60], [61]]. Concurrently, the GSDMD-N terminus can activate caspase-1 [62], promoting the maturation and release of IL-1β and IL-18 via the NLRP3/caspase-1 pathway. The extracellular secretion of IL-18 and IL-1β amplifies the inflammatory response [37,56,57,63].

#### 2.1.3 Other pathways

Beyond caspase-1/11/4/5, caspases-3 and 8 also play significant roles in activating cell pyroptosis [64]. Chemotherapeutic activation of caspase-3 triggers a GSDME-dependent pyroptosis process. Specifically, caspase-3 cleaves GSDME to release the GSDME-N terminal, which then translocates to the plasma membrane and forms pores, resulting in pyroptosis [65,66]. Caspase-8 is known to regulate cell pyroptosis [67], and can induce TNF-α driven pyroptosis under hypoxic conditions by cleaving GSDMC [68]. Moreover, GSDMB is cleaved by lymphocyte-derived granzyme A (GZMA) into its N-terminal fragment, leading to pore formation and ultimately cell swelling and rupture, thereby inducing pyroptosis. Research has shown that interferon-gamma (IFN-γ) up-regulates the expression of GSDMB and enhances pyroptosis [69]. Additionally, NK cells and CTLs can activate caspase-3 through the release of granzyme B (GZMB), which in turn cleaves GSDME to further facilitate extensive pyroptosis [70].

## 3 Applications of pyroptosis activators in tumor immunotherapy

### 3.1 Pyroptosis activators that cause mitochondrial damage

**Fig. 3:**
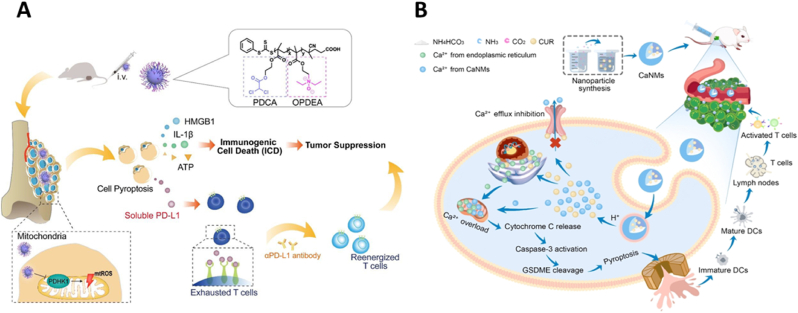
Inducing tumor cells to undergo pyroptosis by mitochondria damage. (A) Mitochondrial targeted OPDEA-PDCA induces cell pyroptosis and enhances tumor inhibition in combination with anti-PD-L1 monoclonal antibody. Reused with permission [74]. Copyright 2022, American Chemical Society. (B) CaNMs activates pyroptosis through mitochondrial Ca^2+^ overload for cancer immunotherapy. Reused with permission [77]. Copyright 2022, Wiley-VCH.

Mitochondria plays pivotal roles in cell survival, proliferation, and has multiple functions such as Ca^2+^ storage, apoptosis, energy production, oxidative stress response, and the tricarboxylic acid (TCA) cycle [71]. Mitochondrial dysfunction is a known critical effector of programmed cell death (PCD) [72]. Pyruvate dehydrogenase kinase 1 (PDHK1), a mitochondrial enzyme, regulates the entry of substrates into the TCA cycle. A reduction in PDHK1 activity is known to initiate PCD, highlighting its potential as a therapeutic target [73]. In this research, Jin et al. [74] developed a mitochondria-targeting strategy using a polymer micelle named OPDEA-PDCA, constructed from the self-assembly of amphiphilic block polymers (OPDEA-*b*-PDCA), to induce pyroptosis by promoting mitochondrial oxidative stress (Fig. 3A). The component tertiary amine-oxide-based zwitterionic poly [2- (*N*-oxide-*N, N*-diethylamino) ethyl methacrylate] (OPDEA) specifically targets mitochondria. Meanwhile, dichloroacetate (DCA), an inhibitor of PDHKs, enhances the mitochondrial oxidative stress, as demonstrated in previous studies. Detailed experimental results revealed that OPDEA-PDCA primarily induced pyroptosis in tumor cells by inhibiting PDHK1, increasing mitochondrial reactive oxygen species (mtROS) production, and activating cytochrome C. Notably, the pyroptosis triggered by OPDEA-PDCA was qualified as immunogenic cell death (ICD), confirmed through the release of immunogenic factors like IL-1β and high mbility group protein 1 (HMGB1), which fostered a pro-inflammatory tumor microenvironment. Moreover, OPDEA-PDCA has been shown to stimulate the secretion of soluble programmed cell death ligand 1 (PD-L1) molecules. Consequently, a combination therapy comprising OPDEA-PDCA and the anti-PD-L1 monoclonal antibody markedly inhibited the growth of osteosarcoma cells and extended T-cell activation.

The capacity of mitochondria to store Ca^2+^ is crucial for cell survival and proliferation. An imbalance between bound and free Ca^2+^ within mitochondria under pathological or toxic conditions leads to the release of cytochrome C, initiating apoptosis [75,76]. The potential of exploiting mitochondrial Ca^2+^ overload to induce pyroptosis in cancer therapy is currently under investigation. Zheng et al. [77] introduced a Ca^2+^ nanomodulators (CaNMs), composed of CaCO_3_ and curcumin (CUR), which induced pyroptosis in cancer immunotherapy via mitochondrial Ca^2+^ overload (Fig. 3B). CaCO_3_ not only served as a carrier for CUR but also helped to elevate Ca^2+^ levels within tumor cells, whereas CUR regulated the mitochondrial Ca^2+^ concentration. This combination caused a significant increase in mitochondrial Ca^2+^, triggering cytochrome C release and subsequent caspase-3 activation, which cleaved GSDME. The N-terminal fragment of GSDME binded to the membrane phospholipids, forming pores that initiate pyroptosis. This event led to the liberation of numerous inflammatory molecules and cellular contents, eliciting a robust anti-tumor immune response. The *in vivo* studies confirming the efficacy and biological safety of CaNMs also demonstrated their role in suppressing tumor growth and lung metastasis, underscoring the potential of mitochondrial Ca^2+^ overload as a therapeutic strategy in tumor immunotherapy.

Furthermore, selective initiation of pyroptosis in cancer cells is essential for effective cancer therapy. Wang et al. [78] identified that a hemicyanine (Cy) fluorophore with an amino group (-NH_2_), referred to as CyNH_2_, could induce pyroptosis to eliminate tumor cells. Because of the prevalent overexpression of NAD(P)H: quinone oxidoreductase isozyme 1 (NQO1) in many cancer types, an NQO1-responsive version of CyNH_2_, named NCyNH_2_, was developed to improve the specificity for targeting tumor cells. In tumor cells rich in NQO1, the near-infrared fluorescence and cytotoxic properties of NCyNH_2_ were reactivated, thereby triggering pyroptosis by accumulating within acitve mitochondria, which successively caused mitochondrial membrane damage, cytochrome C release and caspase-3 activation. The pyroptosis driven by NCyNH_2_ not only killed the tumor cells but also enhanced the antitumor immune responses, making it a potent candidate for immunotherapy. For effective delivery, NCyNH_2_ was incorporated into a nanocarrier composed of an amphiphilic polymer, poly (ethylene glycol)-block-poly (lactic-*co*-glycolic acid) (PEG-*b*-PLGA), to facilitate systemic administration. When combined with the immune checkpoint inhibitor anti-programmed death-1 (αPD-1), this formulation showed significant potential to suppress tumor growth in mice and prevent tumor recurrence.

To further explore the role of mitochondria in pyroptosis, the research by Miao et al. [79] indicated that in the early stages of pyroptosis, GSDMD-NT would first translocate to mitochondria, causing mitochondrial damage before the formation of pores in the cell membrane. This process depends on the translocation of cardiolipin from the inner mitochondrial membrane (IMM) to the outer mitochondrial membrane (OMM) and is independent of proteins like BAX (B-cell lymphoma-2-associated X proten) and BAK (B-cell lymphoma-2 homologous killer) that mediate apoptosis. *In vitro* experiments revealed that GSDMD-NT could directly damage mitochondria, and the mitochondrial damage mediated by GSDMD induced the release of the 3′-5′ exonuclease polyribonucleotide nucleotidyltransferase 1 (PNPT1) into the cytoplasm, leading to widespread mRNA degradation that could exacerbate the occurrence of pyroptosis and amplify downstream inflammatory responses. This study is the first to elucidate the key molecular mechanism by which GSDMD induces mitochondrial damage in the early stages of pyroptosis and the critical "irreversibility" and "signal amplification" functions of mitochondrial damage in the process of pyroptosis.

Therefore, mitochondrial damage leads to the release of cytochrome C or mitochondrial DNA (mtDNA) from the mitochondria into the cytoplasm, which play a key role in pyroptosis, promoting inflammatory responses and cell death. Additionally, GSDMD-mediated mitochondrial damage is considered a key molecular mechanism in the early stages of pyroptosis, playing a crucial role in the amplification of pyroptosis signals and the enhancement of the body's anti-infection/anti-tumor immune responses.

### 3.2 Pyroptosis activators that cause lysosomal disruption

Endosomes and lysosomes, key organelles in cellular metabolism, can degrade various biological macromolecules including proteins, nucleic acids, and polysaccharides. These organelles are pivotal in numerous physiological processes such as cell proliferation, metabolism of proteins and lipids, cell death, and immune responses [80]. Recent research has revealed that targeting lysosomes can trigger diverse PCD pathways, including caspase-independent lysosomal cell death, ferroptosis, autophagy, and pyroptosis [[81], [82], [83], [84]]. Evelyn Ploetz et al. [85] engineered lipid-coated MIL-100 (Fe) metal–organic framework (MOF) nanoparticles (Lip-MOF NPs) composed of Fe^3+^ and trimesic acid, which were specifically designed to transport large amounts of Fe^3+^ efficiently into cells. Upon cellular uptake through grid proteins, the nanoparticles were trafficked to acidic lysosomes, where they underwent reduction by cysteine, resulting in their breakdown and subsequent lysosomal rupture. Meanwhile, this disruption liberated a substantial quantity of Fe^3+^, inducing cell pyroptosis. Detailed mechanistic investigations have demonstrated that the pyroptosis was driven by the activation of caspase-1, which subsequently triggered the activation of GSDMD and IL-1β, ultimately leading to cell swelling and lysis. Furthermore, these nanoparticles have been shown to stimulate the immune system by inducing pyroptosis in primary tumor, suggesting their potential utility in cancer immunotherapy.

**Fig. 4:**
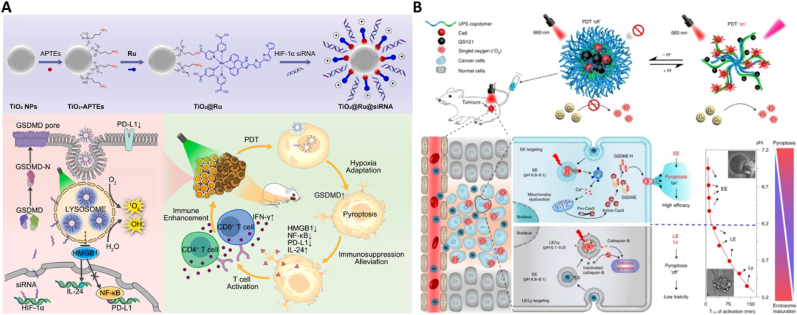
Tumor cell pyroptosis induced by lysosomal disruption (A) TiO_2_@Ru@siRNA could induce Type I and II PDT upon light irradiation, leading to lysosomal damage, HIF-1α gene silencing, and pyroptosis. Reused with permission [86]. Copyright 2022, Elsevier. (B) ANPS library with varies pH transition points to precisely target endosomes at various stages of maturation to modulate cell pyroptosis in a tunable manner. Reused with permission [87]. Copyright 2022, Springer Nature.

Light-induced stimulation can also lead to lysosomal damage, which in turn triggers pyroptotic cell death. Zhou et al. [86] created a TiO_2_@Ru@siRNA nanocomposite through a novel approach by integrating a ruthenium-based photosensitizer (Ru) with TiO_2_ nanoparticles, which were further loaded with hypoxia-inducible factor-1α (HIF-1α) siRNA [90]. Upon exposure to visible light, the TiO_2_@Ru@siRNA nanocomposite initiated Type I and Type II photodynamic reactions that could cause lysosomal damage, release of HIF-1α siRNA, and ultimately the death of oral squamous cell carcinoma (OSCC) cells (Fig. 4A). This method not only mitigated hypoxia but also stimulated pyroptosis through photodynamic therapy (PDT), significantly improving the tumor microenvironment and boosting the anti-tumor immune response, as validated in both the patient-derived xenograft and 4-nitroquinoline-1-oxide (4NQO)-induced rat oral carcinogenesis models. Furtherly, to mitigate the adverse effects of uncontrolled pyroptosis on normal cells, Chen et al. developed acid-activatable nanophotosensitizers (ANPS) with pH transition points ranging from 5.0 to 7.0, covering the entire spectrum of endosomal maturation stages (pH = 5.0 to 7.0) (Fig. 4B). These nanophotosensitizers could precisely target endosomes at various stages of maturation to modulate cell pyroptosis in a tunable manner [87]. This study highlighted that ANPS targeting early endosomes could specifically activate phospholipase C (PLC) signaling on the endosomal membrane and the downstream caspase-3/GSDME pathway, leading to efficient tumor cell pyroptosis. In contrast, ANPS targeting late endosomes/lysosomes could increase lysosomal membrane permeability, resulting in cell apoptosis with less efficiency. These “pyroptosis tuners” could differentiate cell death patterns, demonstrating a significantly greater anti-tumor effect *in vitro* and *in vivo* when targeting early endosomes compared to late stages. The research offers novel perspectives on designing nanomedicines with adjustable pyroptotic activity through precise modulation of endocytic signaling pathways, paving the way for cancer immunotherapy that leverages pyroptotic cell death.

The integrity of the lysosomal membrane is crucial for cell's fate, lysosomal disruption leads to the release of hydrolytic enzymes and other contents from lysosomes into the cytoplasm, triggering a series of cell death signaling pathways, which ultimately result in pyroptosis [88]. Furthermore, autophagy inhibition caused by lysosomal rupture, by blocking the lysosome-mediated massive degradation pathway, improves copper ion overload-mediated cuproptosis and pyroptosis, thus generating a potent antitumor immune response [89]. It is interesting to note that the lysosomes of tumor cells are more fragile than those of normal cells, making them more susceptible to lysosomal rupture and lysosome-dependent cell death [90]. This suggests that strategies targeting lysosome-dependent cell death pathways may be an effective therapeutic strategy for various types of cancer. However, the current understanding of lysosome-dependent cell death is limited, and the molecular targets and mechanisms involved require further in-depth research.

### 3.3 Pyroptosis activators that cause lipid peroxidation of cell membranes

**Fig. 5:**
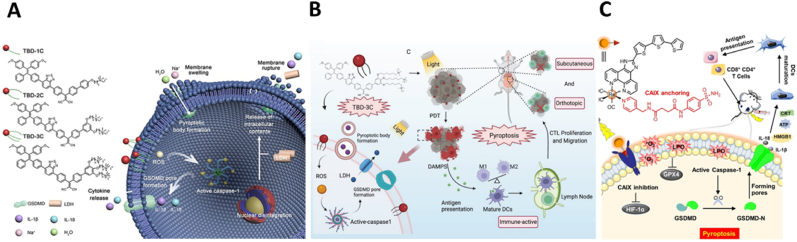
Tumor cell pyroptosis induced by lipid peroxidation of cell membranes. (A) Membrane anchored photosensitizers TBD-1C, TBD-2C, and TBD-3C promote cell membrane lipid peroxidation and induce tumor cell pyroptosis under light irradiation. Reused with permission [98]. Copyright 2021, Wiley-VCH. (B) The antitumor immunotherapy induced by photodynamic pyroptosis of TBD-3C. Reused with permission [103]. Copyright 2022, Wiley-VCH. (C) Membrane anchored photosensitizer CA-Re induces membrane rupture to evoke pyroptosis and anti-tumor immunity upon irradiation. Reused with permission [104]. Copyright 2021, Wiley-VCH.

The cell membrane, considered as protective barrier for cells, maintains cellular integrity and intracellular functions. Its vulnerability has rendered it an emerging target for cancer therapy [[91], [92], [93]]. Recent advancements in membrane-targeting PDT have successfully induced phospholipid peroxidation, enhancing plasma membrane permeability and instability, and in severe cases, causing membrane rupture [[94], [95], [96]]. Furthermore, damage to the plasma membrane has been verified to initiate inflammasome activation and IL-1β release, consequently leading to pyroptosis [97]. Wu et al. developed a range of membrane-anchoring photosensitizers (TBD-1C, TBD-2C, and TBD-3C) characterized by aggregation-induced emission (AIE) to enhance PDT-induced cancer cell pyroptosis [98]. These photosensitizers' cationic side chains improved their solubility in water and affinity for lipid membranes. The series varied by the number of cationic chains linked to a consistent aromatic base to assess their effects on membrane attachment and pyroptosis initiation post-PDT [[99], [100], [101], [102]]. Experimental data revealed a direct relationship between the number of cationic chains and their membrane binding efficiency. TBD-3C, with three cationic chains, showed superior membrane attachment and the highest effectiveness in destroying cancer cells upon light activation. This variant produced reactive oxygen species (ROS) directly at the cell membrane, prompting lipid peroxidation and leading to predominant cell death via pyroptosis (Fig. 5A). Pyroptosis holds significant promise for activating anti-tumor immune mechanisms, largely because it facilitates the liberation of cellular components and inflammatory cytokines. In a subsequent study, Wang et al. [103] demonstrated that pyroptosis, triggered by TBD-3C under light exposure, could effectively counteract the immunosuppressive environment typically seen in pancreatic cancer, thereby enhancing the efficacy of immunotherapy (Fig. 5B). Comprehensive mechanistic investigations revealed that TBD-3C achieved robust anchoring to tumor cell membranes and selectively initiated pyroptosis upon photostimulation. This activation led to the remodeling of the tumor's immunosuppressive landscape, marked by advanced maturation of antigen-presenting cells (APCs), increased T-cell infiltration, and notably, the suppression of tumor progression in both subcutaneous and orthotopic xenograft models.

Similarly, Su et al. [104] formulated a novel rhenium (I)-based photosensitizer, CA-Re, specifically designed to target the transmembrane protein, carbonic anhydrase IX (CAIX) (Fig. 5C). This transmembrane protein is significantly overexpressed in tumors, in stark contrast to its scant presence in normal tissues, with its regulation controlled by HIF-1α [105,106]. The distinctive anchoring capability of CAIX allows CA-Re to maintain prolonged adherence to the cell membrane. Upon exposure to light, CA-Re catalyzed the localized generation of ROS and substantial lipid peroxidation, driving highly effective PDT characterized by its nanomolar phototoxicity. This precision-targeted PDT enhanced membrane permeability, precipitated membrane rupture, and accelerated the release of inflammatory cytokines and DAMPs. Such dynamics bolstered DC activation, maturation, and antigen presentation, igniting T-cell-mediated adaptive immune responses *in vivo*. This cascade of immune activation not only obliterated the primary tumor but also curtailed the proliferation of distant tumors, underscoring the potent immunotherapy of CA-Re. Furthermore, Tang et al. [107] developed a tumor cell membrane-targeting aggregation-induced emission (AIE) photosensitizer that could induce pyroptosis through the synergistic therapy of PDT and PTT. This photosensitizer made by photosensitive dimer (D1) is capable of generating type-I ROS through PDT in hypoxic tumor environments, inducing cell membrane peroxidation mediated pyroptosis, a process that is augmented by its photothermal capabilities. Moreover, the enhanced ICD effect conferred by D1 not only completely eradicated the primary tumor but also stimulated a systemic antitumor immune response, as evidenced by the generation of immune memory, which was reflected in the superior therapeutic outcomes against both solid and metastatic tumors in 4T1 tumor mouse models, which are typically characterized by low immunogenicity.

Therefore, cell membrane-targeting photosensitizers are believed to increase cell membrane permeability, disrupt membrane integrity, induce lipid peroxidation, inactivate membrane-anchored signaling proteins, ultimately leading to cell membrane rupture and intense pyroptosis. In addition, under certain pathological conditions, such as infection, tumor, and some autoimmune diseases, the lipid peroxidation of the cell membrane may play an important role in cell death and tissue damage [108], thus regulating the level of lipid peroxidation in the cell membrane may provide new strategies for the treatment of related diseases.

### 3.4 Pyroptosis activators that cause GSDM improvement

#### 3.4.1 DNA demethylation

Cell death modalities are influenced not solely by caspase activity in apoptosis and inflammation, but also by the specific substrates they hydrolyze [109]. In many tumor cells, the expression of GSDME is significantly reduced due to the methylation of the promoter region of the DFNA5 gene, which encodes GSDME, thereby decreasing the efficiency of pyroptosis induction [110,111]. To counteract this, epigenetic interventions, particularly DNA demethylation, are being explored as a method to reverse DNA hypermethylation in tumor cells [112]. In this study, Zhao et al. [113] devised a biomimetic nanoparticle (BNP) utilizing a poly (lactic-*co*-glycolic acid) (PLGA) core wrapped in cancer cell membrane, encapsulating both indocyanine green (ICG) and the DNA demethylation agent decitabine (DCT). Administered intravenously, this BNP specifically targets and accumulates at the primary tumor site. Upon activation by low-dose laser irradiation, the BNP generates localized hyperthermia, leading to tumor cell membrane disruption. This activation facilitates the release of DCT, enhancing GSDME expression and synergistically promoting pyroptosis. 10.13039/100014337Furthermore, the inflammatory molecules released by tumor cells undergoing pyroptosis fosterDC maturation and T-cell activation within tumor-draining lymph nodes (TDLNs), supporting immunotherapy strategies for both primary and metastatic tumors while minimizing systemic side effects. Similarly, Yu et al. [114] utilized a neutrophil camouflaged stealth nanovehicle (ANP), which is a bovine serum albumin nanoparticle modified with anti-CD11b antibodies and IR820, and loaded with DCT, to achieve photothermal-enhanced pyroptosis for tumor immunotherapy. After systemic administration, the nanovehicle could be efficiently delivered to the tumor site by leveraging the anti-CD11b antibody to target activated neutrophils in the bloodstream. Subsequently, the upregulation of GSDME protein expression caused by the release of DCT, together with the activation of caspase-3 by laser irradiation, triggered pyroptosis. This pyroptotic response not only enhanced the adaptive immune response of the body, modulated the immunosuppressive tumor microenvironment, but is also crucial for effective tumor immunotherapy, thus playing a key role in preventing lung metastasis. Moreover, in another study, the aggregation-induced emission luminogen (AIEgen) and DCT were integrated into the hypoxia-responsive covalent organic frameworks (COFs) to form TD@COFs for pyroptosis-mediated tumor immunotherapy [115]. Owing to the outstanding ability to generate ROS upon PDT and its self-accelerating release mechanism for DCT, TD@COFs could effectively suppress the expansion of the primary tumor and also impede the advancement of metastatic tumors in mice bearing 4T1 tumors.

**Fig. 6:**
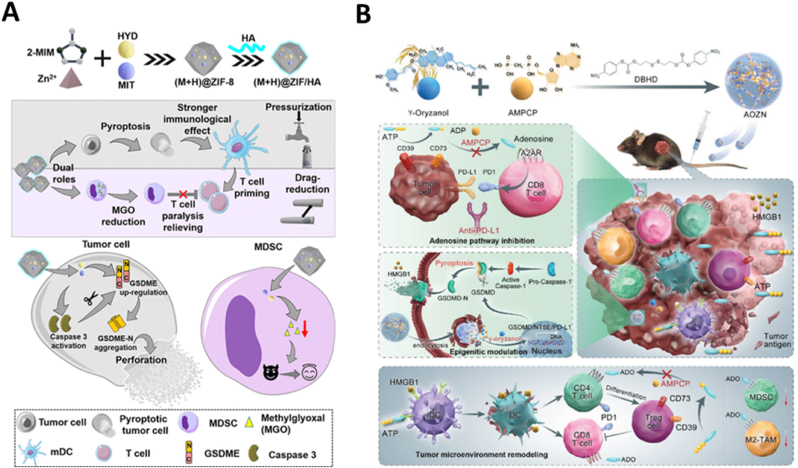
Enhancing intracellular GSDM protein expression through DNA demethylation to induce tumor cell pyroptosis. (A) (M + H) @ZIF/HA exerts chemoimmunotherapeutic ability in tumor cells and MDSCs. Reused with permission [118]. Copyright 2021, American Chemical Society. (B) AOZN utilizes the inhibition of CD73-adenosine and epigenetic modulation to trigger cell pyroptosis for cancer immunotherapy. Reused with permission [127]. Copyright 2021, Wiley-VCH.

Many chemotherapeutic agents activate caspase-3-mediated apoptosis to eliminate tumor cells. However, in the presence of sufficient GSDME, these agents can also initiate the pyroptosis pathway, which additionally triggers immune responses [116,117]. This transformation of apoptosis to pyroptosis in chemotherapy can convert a 'cold' tumor, typically invisible to the immune system, into a 'hot' tumor, which is more susceptible to immune-mediated control. Zhou et al. [118] developed a pH-sensitive ZIF-8 nanoparticle system for the co-delivery of the chemotherapeutic agent mitoxantrone (MIT) and the DNA demethylating agent hydralazine (HYD). To enhance its circulation time, anionic hyaluronic acid (HA) was conjugated to its surface (Fig. 6A). This dual-function nanoplatform not only promotes the apoptosis-to-pyroptosis shift but also disrupts the immune evasion tactics of MDSCs, thereby amplifying the immunotherapeutic impacts of pyroptosis. It has been demonstrated that HYD promotes DNA demethylation, upregulating GSDME protein expression and converting MIT-induced apoptosis into pyroptosis. Concurrently, HYD prevents the formation of methylglyoxal (MGO) in MDSCs, alleviating T cell suppression [119]. Ultimately, this nanoplatform exhibits potent anti-cancer efficacy and establishes a long-term immune memory response against metastatic tumors.

However, recent studies have demonstrated that hypomethylating drugs may increase the expression of immunosuppressive ligands PD-L1/PD-L2, thereby rendering tumors more susceptible to PD-L1 checkpoint blockade therapy [[120], [121], [122], [123], [124]]. Concurrently, these agents may inadvertently activate negative regulatory molecules such as ecto-5′-nucleotidase (CD73), which rapidly converts adenosine triphosphate (ATP) into immunosuppressive adenosine (ADO) within tumors, potentially facilitating local tumor progression and distant metastasis [125,126]. Combining epigenetic modifications with CD73 inhibitors could therefore potentiate the effectiveness of anti-PD-L1 treatments. In this research, Xiong et al. [127] engineered glutathione (GSH)-responsive prodrug nanomicelles (AOZN), integrating γ-oryzanol (Orz) for DNA methyltransferase inhibition and α, β-methylene adenosine 5′ diphosphate (AMPCP) for CD73 inhibition, bound together through disulfide bonds and hydroxyl cross-linking (Fig. 6B). In the GSH-rich tumor microenvironment, AOZN prodrugs disintegrate specifically, minimizing harm to healthy tissues. Released Orz boosts tumor immunogenicity by upregulating GSDMD and PD-L1 expressions, while released AMPCP curtails ADO accumulation by inhibiting CD73. Together, these actions synergistically modulate the immunosuppressive tumor environment and amplify the impact of anti-PD-L1 therapy, as evidenced by robust antitumor immune responses observed in the B16F10 mouse model.

#### 3.4.2 Others

**Fig. 7:**
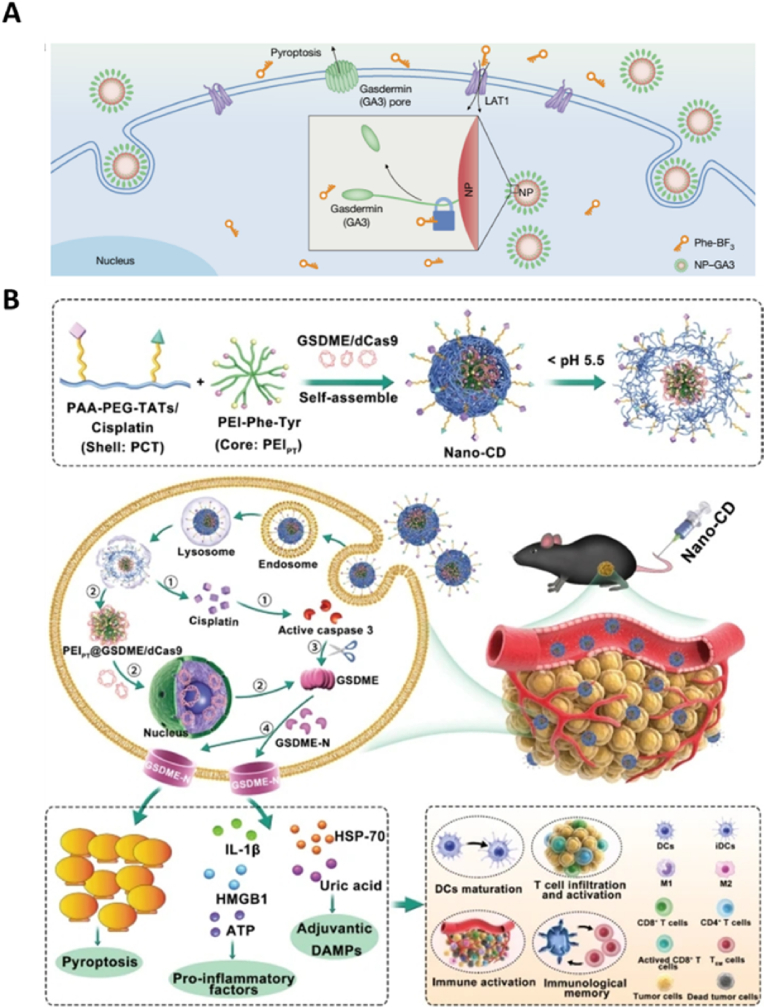
Inducing cell pyroptosis by improment of GSDM proteins in tumor cells. (A) A bioorthogonal chemical system uses Phe-BF_3_ desilylation to release GSDM for achieving controllable activation of cell pyroptosis in tumors. Reused with permission [18]. Copyright 2020, Springer Nature. (B) Nano-CD scaffold initiates pyroptosis induced immune response through the self supply of GSDME. Reused with permission [128]. Copyright 2023, Springer Nature.

GSDM proteins, the key executors of pyroptosis, become activated within tumor cells, where their N-terminal domains interact with membrane phospholipids to perforate the cell membrane, leading to lytic cell death and a potent inflammatory response. Thus, enhancing GSDM protein levels in tumor cells is an effective strategy to induce pyroptosis. Wang et al. [18] have developed a bioorthogonal chemical system for intracellular delivery of GSDM proteins (Fig. 7A). This system utilizes the cancer imaging probe phenylalanine trifluoroborate (Phe-BF_3_), which specifically targets tumor cells, undergoes desilication, and cleaves a silyl ether-containing linker. This desilylation reaction catalyzed by Phe-BF_3_ allows for controlled drug release from antibody-drug conjugates within the tumor cells. Leveraging this bioorthogonal approach, the team effectively initiated *in situ* and controllable activation of pyroptosis in tumors. Further investigations revealed that inducing pyroptosis in as few as 15 % of tumor cells can significantly modulate the tumor immune microenvironment, activate robust T-cell-mediated anti-tumor responses, and eradicate 4T1 breast tumor grafts entirely. This pioneering study opens up novel avenues for the development of cancer immunotherapy agents, positioning GSDM family protein agonists as a promising new class of anti-cancer therapeutic agents.

Considering the challenges associated with the stability and bioavailability of externally delivered GSDM proteins, developing strategies for intracellular self-production of GSDM is particularly promising. By integrating nuclease-inactivated CRISPR-associated protein 9 (Cas9) with a transcriptional activator, the CRISPR/dCas9 system can harness a tumor cell's own genetic machinery to synthesize biologically active GSDM proteins, thereby ensuring a continuous internal supply. Wang et al. [128] utilized this innovative approach for pyroptosis-based immunotherapy by creating a Nano-CRISPR scaffold (Nano-CD) (Fig. 7B). This system combines an amino acid-modified cationic core with a versatile copolymer to co-deliver CRISPR/dCas9 and cisplatin. After cellular uptake, Nano-CD responds to the acidic intracellular environment to release its contents as needed. The CRISPR/dCas9 component activates the tumor's own GSDME gene, while cisplatin induces caspase-3 activation, which cleaves the newly synthesized GSDME, triggering potent pyroptosis. This process not only results in significant tumor cell destruction but also enhances immune responses by facilitating DC maturation, antigen presentation, and T-cell activation, ultimately leading to immunogenic tumor cell death. When used in conjunction with checkpoint blockade therapy in a malignant melanoma model, Nano-CD demonstrates profound therapeutic benefits, significantly reducing tumor recurrence and metastasis.

In summary, DNA demethylation and intracellular delivery of GSDM protein can effectively increase the content of GSDM proteins within cells. GSDM proteins are the key executors in the process of pyroptosis, and an increase in their content can enhance the cells' response to inflammatory signals, thereby effectively triggering pyroptosis and enhancing the immune response against tumors. However, it should be noted that the increase in GSDM content should be carried out under strict control to avoid excessive inflammatory responses and unnecessary tissue damages.

### 3.5 Pyroptosis activators that cause DNA damage

**Fig. 8:**
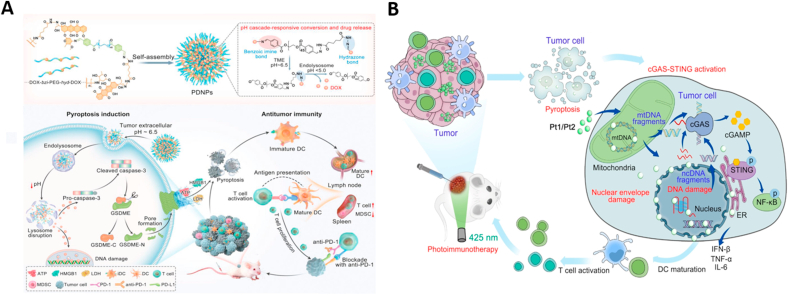
Inducing DNA damage within cells to trigger cell pyroptosis. (A) PDNPs undergo cascade transformation under acidic pH response and release chemotherapy drugs to induce pyroptosis. Reused with permission [133]. Copyright 2022, Wiley-VCH. (B) Pt1 and Pt2 sequentially damage mtDNA, nuclear membrane, and ncDNA under light irradiation, activating the cGAS-STING pathway and pyroptosis to induce a strong anticancer immune response. Reused with permission [137]. Copyright 2022, Wiley-VCH.

Chemotherapy drugs disrupt DNA synthesis and repair mechanisms, inhibit DNA replication, and block cell division, which curtails the proliferation and dissemination of cancer cells. Studies have identified that multiple chemotherapy agents can trigger caspase-3-mediated pyroptosis in tumor cells with elevated levels of GSDME, thereby boosting the immunogenicity of these cells [65,129,130]. Despite the potential of chemotherapy-induced pyroptosis to enhance immunotherapy outcomes, biological barriers during drug delivery can significantly diminish the effectiveness of chemotherapy, sometimes preventing the drugs from achieving their desired effects or causing severe side effects [131,132]. To overcome these challenges, Liang et al. [133] designed a drug-polymer hybrid supramolecular nanoprodrug (PDNP) that exhibits stepwise size shrinkage and cascade-activation properties (Fig. 8A). This PDNP can penetrate deeply into tumors and release chemotherapy drugs precisely within tumor cells. Comprising multiple drug-polymer units linked by acid-cleavable linkers, PDNPs at physiological pH exhibit 'stealth' characteristics due to PEGylation, allowing them to accumulate effectively at tumor sites. They respond sequentially to the acidic tumor microenvironment and even more acidic intracellular conditions, such as in lysosomes, to ensure deep tumor penetration and targeted delivery of the chemotherapy drug doxorubicin (DOX) to the nucleus, where it provokes GSDME-mediated pyroptosis. Moreover, Yin et al. [134] introduced a novel gold nanorod-engineered macrophage system, modified via liquid nitrogen freezing, that functions as a near-infrared light controllable bioinspired killing (BIK) system. This system, by inducing pyroptosis through ultralow-dose chemotherapy, stimulates potent anti-tumor immune responses. The BIK system minimizes drug consumption to less than one-thirtieth of the typical dose *in vitro* and exhibits effective tumor targeting and penetration *in vivo*. It was demonstrated that this approach could induce pyroptosis and effectively regress tumors under extremely low doses of DOX without damaging major organs. Notably, the combination of ultralow dose DOX and αPD-1 therapy significantly suppressed the growth of large-volume EMT6 tumors in advanced stages. This research offers valuable insights into designing nano-assisted systems that activate anti-tumor immunity with minimal stimuli, underscoring the potential of ultralow dose chemotherapy to induce effective anti-tumor immunity through pyroptosis.

Platinum-based chemotherapy agents often suffer from limited selectivity, high toxicity to normal cells, and a propensity for inducing drug resistance [135,136]. Consequently, the development of platinum complexes with enhanced selectivity and novel mechanisms of action is of considerable interest. Ling et al. [137] have synthesized two photo-activated platinum complexes, Pt1 and Pt2, which activate the cGAS-STING pathway and simultaneously induce cancer cell pyroptosis, eliciting strong anticancer immune responses both i*n vitro* and *in vivo* (Fig. 8B). Structurally, Pt1 and Pt2 incorporate an alkylated triphenylamine ligand L, drawing similarities to cisplatin and transplatin, respectively. Upon light irradiation, these complexes inflict continuous damage to mitochondrial DNA (mtDNA), the nuclear envelope, and nuclear DNA (ncDNA), triggering the cGAS-STING pathway and promoting a pyroptosis-mediated immune response against cancer. Experimental findings i*n vivo* demonstrated that Pt1 and Pt2 not only suppress tumor growth but also stimulate the release of cyclic GMP-AMP (cGAMP) and pro-inflammatory cytokines, thereby increasing the populations of CD8^+^ and CD4^+^ T cells. This groundbreaking study introduces the first small molecule-based photoactivators that can both activate the cGAS-STING pathway and induce pyroptosis, offering a novel strategy for cancer immunotherapy that leverages both pathways to combat tumor growth effectively.

In summary, chemotherapy drugs induce DNA damage, which in turn activates caspase-3 and cleaves the GSDME protein, leading to pyroptosis. However, this pathway of pyroptosis is also one of the important reasons for the side effects of some chemotherapy drugs: in various human cells, cells expressing high levels of GSDME can undergo GSDME-dependent pyroptosis under the action of chemotherapy drugs [65]. Therefore, it is particularly important to develop pyroptosis-inducing strategies with excellent tumor cell targeting.

### 3.6 Pyroptosis activators that cause inhibition of oncogenic signaling pathway

**Fig. 9:**
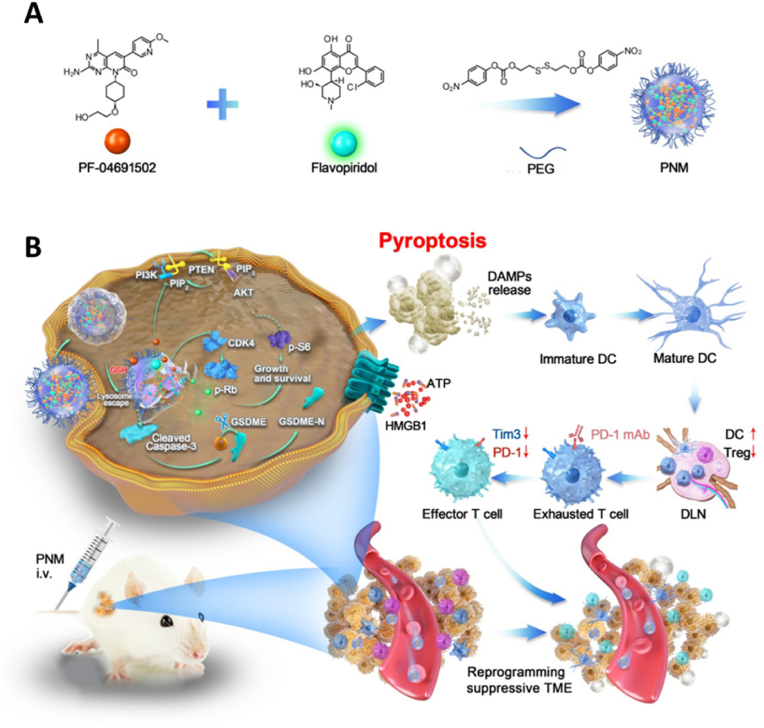
Initiate pyroptosis by inhibiting the oncogenic signaling pathway. (A) Constuction of a GSH-responsive nanomicelle PNM to co-deliver PI3K/mTOR inhibitor and CDK inhibitor. (B) PNM effectively induces pyroptotic cell death and enhances antitumor immunotherapy by strategically blocking the PI3K/mTOR and CDK pathways**.** Reused with permission [147]. Copyright 2022, Elsevier.

Phosphoinositide 3-kinase (PI3K) and cyclin-dependent kinase (CDK) pathways are pivotal in regulating tumor growth and evading immune surveillance [138,139]. Often mutated in various cancers, PI3K triggers the PI3K/Akt/mTOR pathway, reducing the effectiveness of T-cell mediated responses by suppressing the activity of tumor-specific T cells and hindering their infiltration into tumors [[140], [141], [142]]. Moreover, the CDK4/6 pathway has been associated with reduced efficacy of immune checkpoint inhibitors due to its role in immune exclusion [143]. Simultaneous activation of the CDK-Rb pathway may further diminish the impact of PI3K inhibitors [144]. Recent studies suggest that the synergistic inhibition of PI3K and CDK4/6 could reverse drug resistance, promoting tumor regression [145,146]. In this regard, Yang et al. [147] proposed that dual inhibition of PI3K/mTOR and CDK pathways via small molecule drugs could promote caspase-3-induced pyroptosis in tumor cells, thereby enhancing the effectiveness of ICB therapies. They devised a prodrug nanomicelle (PNM), which integrates a PI3K/mTOR inhibitor (PF-04691502, PF) and a broad-spectrum CDK inhibitor (flavopiridol, Flav) within a GSH-responsive nanoplatform (Fig. 9A). This platform ensures targeted delivery and controlled drug release within the tumor microenvironment, characterized by high GSH levels. The PNM facilitates the induction of GSDME-mediated pyroptosis, initiating strong antitumor immune responses. When used in conjunction with αPD-1 therapy, the PNM significantly improves treatment outcomes and prolongs survival, offering a novel and potent approach to cancer immunotherapy (Fig. 9B). As a result, the inhibition of oncogenic signaling pathways may promote the clearance of tumor cells by activating signaling pathways associated with pyroptosis. However, the specific mechanisms of this process may vary depending on factors such as cell type, types and concentrations of drugs, and further research is needed to clarify its potential in cancer treatment.

### 3.7 Pyroptosis activators that cause ROS stress

ROS are pivotal in regulating cell proliferation and differentiation. Maintaining a balance between ROS production and scavenging is essential for the dynamic equilibrium of redox reactions within cells [148]. The increase in ROS levels, depending on their magnitude and the specific conditions within cells, may trigger different types of cell death, including apoptosis and pyroptosis, as well as other forms such as necrosis and ferroptosis [149]. Apoptosis is a programmed form of cell death that typically does not involve an inflammatory response. When the level of ROS within cells is moderate, it can participate in the regulation of the apoptotic process by activating signaling molecules in the apoptotic pathway, such as caspase-3, caspase-8, and caspase-9. When the level of ROS within cells significantly increases, especially in the case of activated inflammasomes, it may activate caspase-1 to trigger pyroptosis. Furthermore, in some cases, an excessive amount of ROS can also lead to necrosis, which is a disorderly form of cell death usually associated with cellular structural damage and an inflammatory response. Lastly, ferroptosis is a specific form of cell death associated with iron metabolism disorders and lipid peroxidation, and it may also be related to changes in ROS levels. The strategic manipulation of ROS has emerged as a promising approach in oncological treatments.

#### 3.7.1 Phototherapy

The photon-mediated biomedical engineering technologies, such as photothermal therapy (PTT) and photodynamic therapy (PDT), have made significant progress in tumor treatment and diagnosis in recent years [[150], [151], [152], [153], [154]]. These phototherapies have the characteristics of non-invasiveness and spatiotemporal control [155,156]. In the process of PDT, ROS with cytotoxic properties is generated by photosensitizers when exposed to light, whereas, during PTT, photothermal agents effectively convert the absorbed light energy into heat energy. PDT can effectively trigger the activation of the inflammasome by generating ROS, leading to pyroptosis, while PTT can intensify this process [157]. Xiao et al. [158] designed a nano-prodrug with dual responsiveness to ROS/GSH in the tumor microenvironment, which induces tumor cell specific pyroptosis by chemo-PDT therapy. Specifically, the engineered nano micelle system (MPEG-CPPA-*b*-P(M4)@SPTX/P18, abbreviated as MCPP) was formed by loading a disulfide functionalized dual paclitaxel prodrug (SPTX) and photosensitizer purpurin 18 (P18) through a self-assembly system of ROS-sensitive amphiphilic block polymers. This nanomicelle significantly improves the tumor targeting ability of traditional chemotherapy drugs and photosensitizers, avoiding systemic toxic side effects. After the enrichment of MCPP in the tumor microenvironment, ROS/GSH dual responses could occur to promote drug release. ROS generated by P18 after laser irradiation together with the chemotherapy drug PTX could jointly induce rapid and persistent pyroptosis by upregulating GSDME in tumor cells. This could subsequently lead to the release of inflammatory factors and tumor associated antigens (TAA), activation of antigen-presenting cells, initiation of adaptive immunity, and ultimately the formation of immune memory. In combination with ICB therapy, MCPP markedly improves therapeutic outcomes, promoting tumor regression and preventing recurrence. Similarly, Zhou et al. [159] exploited the elevated GSH levels within the tumor microenvironment to develop a targeted responsive prodrug, CANPs. This formulation induces ROS-mediated pyroptosis by integrating the heat-shock protein 90 inhibitor tanespimycin (17-AAG) with the photosensitizer chlorin e6 (Ce6). Additionally, 17-AAG bolsters the efficacy of αPD-1 therapy by diminishing the presence of MDSCs [160,161]. In the 4T1 breast cancer mouse model, CANPs enhanced T-cell infiltration, reduced MDSCs, and effectively suppressed both primary and metastatic tumors. This not only amplified the αPD-1 response but also significantly prolonged survival. Consequently, these tumor microenvironment-responsive prodrugs that trigger pyroptosis offer a promising avenue for augmenting immunogenic PDT.

**Fig. 10:**
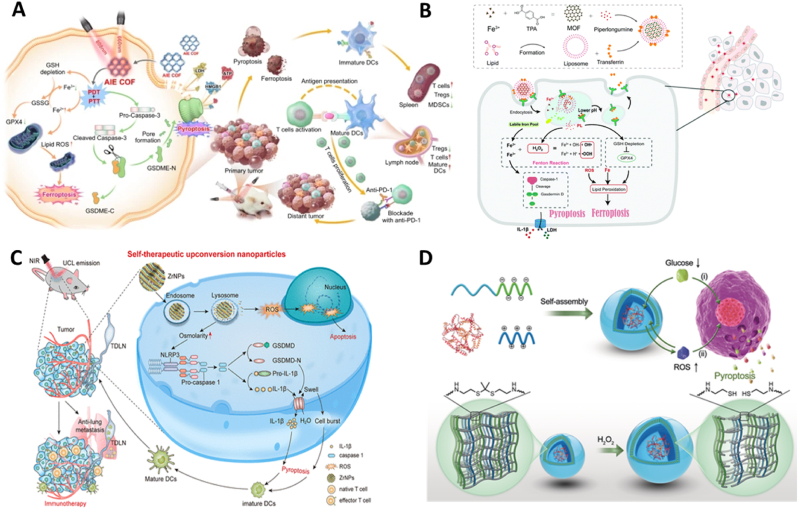
Different strategies to increase intracellular ROS to promote cell pyroptosis. (A) COF-919 integrating planar and twisted AIEgens motifs triggers dual mechanism of tumor pyroptosis and ferroptosis after phototherapy. Reused with permission [162]. Copyright 2023, Springer Nature. (B) Tf-LipoMof@PL increases iron and ROS levels to achieve ferroptosis/pyroptosis dual induction combined therapy. Reused with permission [175]. Copyright 2021, Royal Society of Chemistry. (C) ZrNPs cause a surge in intracellular osmotic pressure by disrupting the homeostatic environment, leading to ROS mediated cell pyroptosis. Reused with permission [185]. Copyright 2021, American Chemical Society. (D) ROS-responsive GOD-loaded nanoreactors with self-enhancing catalytic glucose oxidation achieve pyroptosis-mediated ICD. Reused with permission [188]. Copyright 2020, Wiley-VCH.

Zhang et al. [162] engineered a covalent organic framework (COF-919) incorporating both planar and twisted aggregation-induced emission luminogens (AIEgens) motifs (Fig. 10A). This innovative structure enhances near-infrared light absorption, boosts photothermal conversion efficiency, and extends ROS generation post-PTT. The unique properties of COF-919 facilitate dual induction of tumor pyroptosis and ferroptosis, leading to significant inflammatory responses and strengthened anti-tumor immunity. Mechanistic studies revealed that the COF's effective photothermal activity and ROS generation catalyze lipid peroxidation, deplete GSH, and suppress GSH peroxidase 4 (GPX4) expression, thereby triggering GPX4-related ferroptosis. The resultant pyroptosis and ferroptosis from COF-919 treatment not only release a substantial amount of cellular contents but also stimulate robust immune responses. This effect enhances T-cell infiltration while diminishing regulatory T cells (Tregs) and MDSCs within the tumor microenvironment. Importantly, COF-919 significantly bolsters the efficacy of αPD-1 immune checkpoint blockade therapy, showing a synergistic effect in preventing tumor metastasis and recurrence, achieving over 90 % inhibition of tumor growth and an 80 % therapeutic efficacy rate.

Due to the hypoxic nature of solid tumor microenvironments (*p*O_2_< 5 mm Hg) [163], it poses a challenge for O_2_ dependent PDT [164]. Yu et al. [165] eveloped a low O_2_-dependent photo triggered photocatalytic superoxide radical (O_2_^−•^) generation system (NI-TA) that could activate the caspase-3/GSDME pathway in cancer cells under hypoxic conditions, thereby inducing pyroptosis. In the design of NI-TA, covalently introducing electron donor π-bridge groups into fluorophore skeleton with electron-accepting capability allows for modulation of triplet-ground state splitting energy to promote O_2_^−•^ production. Mechanism studies have confirmed that NI-TA could trigger oxidative stress in tumor cells with low O_2_ levels through photon induced O_2_^−•^ production, superoxide dismutase (SOD) mediated dismutation reactions, and Haber Weiss/Fenton reactions to form hydroxyl radicals (·OH) [166]. Under the condition of light irradiation, NI-TA successfully inhibited the proliferation of hypoxic T47D (human breast cancer cell line) cells and 3D multicellular spheroids (MCSs), which proved the potential of light induced pyroptosis in cancer treatment.

#### 3.7.2 Chemodymic therapy

Chemodynamic therapy (CDT), leveraging endogenous hydrogen peroxide (H_2_O_2_) within tumors to generate cytotoxic ROS via metal-catalyzed Fenton reactions, operates independently of external energy sources and ambient oxygen levels, thereby promoting inflammatory responses and pyroptosis [[167], [168], [169]]. However, the efficacy of CDT is often constrained by insufficient intracellular H_2_O_2_ levels [170]. To overcome this limitation, Zhang et al. [171] innovated by doping COFs with different metals (Cu, Fe, Ni), creating variants such as COF-909-Cu, COF-909-Fe, and COF-909-Ni. These modifications enhanced the optical properties and mimicked enzymatic functions, thereby improving catalytic performance to disrupt H_2_O_2_ homeostasis and effectively enhance pyroptosis. Specifically, these COFs emulate the activities of superoxide dismutase (SOD), peroxidase (POD), and GSH peroxidase (GPx), enhancing ROS production and facilitating GSH depletion. Among these, COF-909-Cu was particularly effective, significantly inducing GSDME-dependent pyroptosis and bolstering anti-tumor immunity *in vivo*. Moreover, COF-909-Cu markedly alters the tumor microenvironment, reducing populations of immunosuppressive cells while increasing memory T cells, synergistically improving the efficacy of αPD-1 checkpoint blockade therapy. This approach represents a significant advancement in employing CDT for cancer immunotherapy, offering notable potential to impact tumor metastasis control and recurrence prevention effectively.

Ferroptosis and pyroptosis are non-apoptotic forms of cell death that offer new avenues for cancer therapy. Despite their potential, therapies leveraging both pathways simultaneously remain underexplored. Studies indicate that elevated intracellular iron and ROS levels can trigger both ferroptosis and pyroptosis [[172], [173], [174]]. In response, Xu et al. [175] developed a dual-induction nanodelivery system, Tf-LipoMof@PL, which encapsulates the ferroptosis inducer piperlongumine (PL) within iron-containing metal-organic frameworks (MOFs) coated with transferrin-modified DOPE pH-sensitive lipid layers (Fig. 10B). This system exploits transferrin's ability to mediate iron uptake via endocytosis through the transferrin receptor (TfR), effectively enhancing intracellular iron levels. Simultaneously, PL contributes to H_2_O_2_ production, thus amplifying lethal ROS generation through the Fenton reaction. Experimental evidence suggests that this increase in iron and ROS not only initiates ferroptosis and pyroptosis but also reduces GSH, further driving ferroptosis via GPX4 inhibition and lipid peroxidation (LPO) [176]. Demonstrating significant anticancer efficacy in a xenograft mouse model, the Tf-LipoMof@PL system illustrates the promising potential of combining ferroptosis and pyroptosis in cancer treatment.

#### 3.7.3 Imbalance of intracellular osmolarity

Exogenous ROS in tumors is predominantly generated by the photodynamic action of photosensitizers or the Fenton reaction involving transition metal ions and hydrogen peroxide (H_2_O_2_) within the mildly acidic tumor microenvironment [167,[177], [178], [179]]. Nonetheless, the efficacy of these ROS-generating methods is hampered by several factors: the limited penetration depth of therapeutic light, the low intracellular concentrations of O_2_ and H_2_O_2_, and the stringent pH requirements (pH 3–4) for optimal Fenton reactions [170,180,181]. Consequently, there is a pressing need to develop innovative strategies to enhance intracellular ROS levels, thus improving the effectiveness of ROS-mediated therapies. Lanthanide-doped upconversion nanoparticles (UCNPs) possess superior optical properties and are frequently utilized in various medical applications due to their unique capabilities [[182], [183], [184]]. Ding et al. [185] innovatively employed biodegradable K3ZrF7: Yb/Er UCNPs (ZrNPs) (Fig. 10C), not just as carriers but as therapeutic agents themselves, demonstrating for the first time their capability to induce pyroptosis and enhance cancer immunotherapy. In cellular environments, ZrNPs dissolve to release significant quantities of K^+^ and [ZrF7]^3−^ ions, disrupting cellular ionic balance and escalating intracellular osmotic pressure. This elevation in osmotic pressure increases oxidative stress and ROS levels, subsequently activating NLRP3 inflammasomes and caspase-1, which promote GSDMD-mediated pyroptosis and IL-1β release. *In vivo* studies highlight ZrNPs’ potent immunomodulatory effects, facilitating DC maturation and proliferation of effector-memory T cells, thus markedly inhibiting tumor growth and lung metastasis.

Additionally, Liu et al. [186] developed PNSO nanoparticles by modifying peroxydisulfate nanoparticles (Na_2_S_2_O_8_) with lecithin and polyethylene glycol phospholipids. These nanoparticles degrade *in situ*, releasing Na^+^ and S_2_O_8_^2−^ ions, the latter converting into highly toxic and long-lived ^•^SO_4_^−^ radicals, independent of oxygen, H_2_O_2_, and pH levels in the tumor microenvironment. The resultant ROS storm and osmotic pressure surge from PNSO nanoparticles cause tumor cell rupture, inducing effective pyroptosis and stimulating robust anti-tumor immunity. These nanoparticles not only alleviate primary tumors through combined ROS and osmotic pressure mechanisms but also modulate tumor immunosuppression and trigger systemic anti-tumor responses, thus effectively preventing tumor metastasis and recurrence. Similarly, Ding et al. [187] utilized microemulsion method to prepare alkalescent sodium bicarbonate nanoparticles (NaHCO_3_ NPs) modified with 1,2-distearoyl-sn-glycero-3-phosphate ethanolamine polyethylene glycol (DSPE-PEG). The resulting NaHCO_3_ NPs were capable of modulating lactic acid metabolism via an acid-base neutralization process, effectively counteracting the mildly acidic conditions that create an immunosuppressive tumor microenvironment. Moreover, the release of a large number of Na ^+^ ions from NaHCO_3_ NPs, inducing a sharp increase in intracellular osmotic pressure, which in turn activated the pyroptosis pathway, significantly inhibiting the growth of primary and distant tumors and tumor metastasis, demonstrating enhanced antitumor immune efficiency.

#### 3.7.4 ROS generators

Li et al. [188] developed innovative ROS-responsive nanoreactors based on polyion complex vesicles (PICsomes), incorporating stimulus-responsive junctions into their cross-linked membrane networks. These nanoreactors are engineered using glucose oxidase (GOD)-loaded PICsomes, constructed from poly([2-[[1-[(2-aminoethyl) thio]-1-methylethyl] thio] ethyl]-α, β-aspartamide) (PATK) and PEG-*b*-poly (α, β-aspartic acid) (PEG-*b*-PAsp), functioning as polycation and polyanion chain segments, respectively (Fig. 10D). Upon exposure to H_2_O_2_, the ROS-responsive bonds in the vesicles gradually degrade, decreasing the membrane's crosslinking density and transitioning the membrane from hydrophobic to hydrophilic, which enhances vesicle swelling and permeability, thus improving the efficiency of catalytic glucose oxidation. Significantly, these vesicles maintain structural integrity throughout the transformation, shielding GOD from adverse environmental impacts and sustaining its activity over extended periods. This capability enables the vesicles to induce glucose deprivation and oxidative stress in tumor cells, leading to cell death predominantly by pyroptosis. This mode of cell death not only eliminates tumor cells but also promotes a robust anti-tumor immune response, evidenced by the release of pro-inflammatory cytokines and immunostimulatory factors.

In summary, ROS plays a key role in the induction and regulation of pyroptosis by modulating the cellular redox balance. ROS can activate the inflammasome, especially the NLRP3 inflammasome, thereby activating caspase-1. The activated caspase-1 can cleave GSDMD, generating an N-terminal fragment of GSDMD with membrane-punching activity. These fragments are translocated to the cell membrane, forming pores that compromise the integrity of the cell membrane, allowing the leakage of cellular contents into the extracellular environment, triggering an inflammatory response, and ultimately leading to cell pyroptosis. Additionally, ROS generated by phototherapy can also induce pyroptosis through the caspase-3/GSDME pathway.

## 4 Summary and prospect

Pyroptosis represents a distinct form of PCD characterized by cell rupture and a potent inflammatory response. Recent research has significantly advanced our understanding of pyroptosis, elucidating its mechanisms, pathological impacts, and its specific role in oncogenesis and cancer therapy. The induction of pyroptosis in tumor cells facilitates the release of antigens, DAMPs, and cytokines, markedly altering the tumor immune microenvironment. This alteration can transform tumors from immunologically "cold" to "hot", enhancing the efficacy of immunotherapies [189,190]. Moreover, the increased expression of GSDM proteins promotes the recruitment of immune cells, including M1 macrophages, CD4^+^, and CD8^+^ T lymphocytes, thereby heightening the responsiveness to ICB treatments. Integrating pyroptosis induction strategies with ICB represents a promising avenue for cancer therapy.

The role of pyroptosis in cancer is multifaceted, often described as a "double-edged sword" within the tumor immune system [34]. While the inflammatory vesicles and cytokines released during pyroptosis can provoke chronic inflammation, they may also foster a microenvironment conducive to tumor growth and metastasis, thereby potentially accelerating tumor progression. Furthermore, pyroptosis has been linked to poor patient outcomes, such as in cases where caspase-8/GSDMC-mediated pyroptosis correlates with adverse prognoses in breast cancer patients. Additionally, pyroptosis is implicated in several detrimental effects associated with cancer treatments, notably cytokine release syndrome following CAR-T cell therapy [[191], [192], [193], [194]]. Therefore, by exploring the effects of pyroptosis on the tumor immune microenvironment, investigating the relationship between the underlying mechanisms in the process of pyroptosis and tumors, the inhibitory effects of pyroptosis on tumors can be more effectively utilized and its promotional effects on tumors can be prevented. In addition, the combination of more systematic clinical research can determine the specific role of pyroptosis in the treatment of different cancers and effectively avoid the adverse effects of treatment.

Furthermore, pyroptosis may cause damage to normal healthy tissues leading to systemic adverse effects. The low bioavailability of some small molecule pyroptosis inducers, insufficiently deep penetration into tumors and lymph nodes, and the immunosuppressive microenvironment also pose limitations to immunotherapy. Consequently, developing effective pyroptosis inducers that specifically target tumor cells is essential. Utilizing nano-intelligent materials to initiate pyroptosis offers a promising and innovative direction for enhancing antitumor immunotherapy. Integrating specific antibodies or chemokine receptors on the surface of nanomaterials loaded with pyroptosis inducers can enhance their navigation within the tumor microenvironment, facilitating more precise targeting of cancer cells. Intelligent nanoreactors capable of responding to internal and external stimuli have the advantages of prolonging blood circulation time, controlling drug release and reducing toxic side effects. These reactors can not only specifically trigger tumor cell pyroptosis, but also eliminate the primary tumor, activate a strong systemic anti-tumor immune response, generate immune memory effects, and inhibit distant tumor metastasis [195]. However, all reported nanomedicines that cause pyroptosis are based on *in vitro* cellular and animal experiments. These drugs have not yet moved to the clinical trial stage. At present, there are several issues that remain unresolved. Firstly, as an artificially synthesized material, nanomaterials need to overcome the difficulty of being recognized and cleared by the immune system in the blood circulation. Secondly, the biosafety of nanomaterials before clinical translation needs to be carefully assessed by unified quantitative indicators. Thirdly, there is a lack of powerful means to achieve the integration of nanomaterial diagnosis and treatment. Finally, the synthesis and cost of nanomaterials are also important factors affecting their application and translation.

Although the development of nanotechnology provides hope for inducing tumor cell pyroptosis, the tumor limitations caused by the downregulation of GSDM protein in many tumors make it difficult to effectively trigger pyroptosis to kill the tumor [196]. The epigenetic mechanism plays an important role in the regulation of pyroptosis. Epigenetic therapy can upregulate the expression of specific proteins, such as upregulating GSDME and ORZ levels using DNA methyltransferase inhibitor DAC, as well as upregulating GSDMD levels in melanoma [129,196]. Therefore, elucidating the epigenetic mechanism of pyroptosis and developing nanomedicines involving epigenetics may be a hot topic for future research.

Further exploration of how pyroptosis regulates immune cells and responses is vital for enhancing cancer treatment efficiency. Investigating interactions between pyroptosis and immune checkpoints could deepen our understanding of immunotherapy mechanisms. Additionally, systematically comparing immune outcomes across different cell death types, including pyroptosis, and elucidating their molecular mechanisms may reveal their potential as clinical therapeutic targets.

## CRediT authorship contribution statement

**Xin Bao:** Writing – original draft. **Mengmeng Sun:** Resources. **Lingfei Meng:** Resources. **Hong Zhang:** Writing – review & editing. **Xuan Yi:** Writing – review & editing. **Peng Zhang:** Writing – review & editing.

## Declaration of competing interest

The authors declare that they have no known competing financial interests or personal relationships that could have appeared to influence the work reported in this paper.

## Data Availability

Data will be made available on request.

## Abbreviations

AMPK: AMP activated protein kinase
Bcl-2: B-cell lymphoma 2
DAMPs: danger-associated molecular patterns
GSDM: gasdermin
HSPs: heat shock proteins
PAMPs: pathogen-associated molecular patterns
TNF-α: tumor necrosis factor-alpha
TRAIL: TNF-related apoptosis-inducing ligand;
ULK1: UNC-51 like kinase 1
VPS34: Vacuolar protein sorting 34
PRR: pattern recognition receptor
IL 1β: interleukin—1β
IL 18: interleukin-18
GSDMD: gasdermin D
HMGB1: high mbility group protein 1
LDH: lactate dehydrogenase
\LPS: lipopolysaccharide;
NK cells: natural killer cells
CTLs: cytotoxic T lymphocytes
GzmB: Granzyme B
GzmA: Granzyme A
GSDMC: gasdermin C
GSDME: gasdermin E
GSDMB: gasdermin B
PDHK1: Pyruvate dehydrogenase kinase 1
ROS: reactive oxygen species
DC: dendritic cells
PLC: phospholipase C
CRT: calreticulin
IFN-γ: interferon—γ
MDSCs: myeloid-derived suppressor cells
Treg: Regulatory T cells
PD-L1: programmed cell death 1 ligand 1
ICD: immunogenic cell death
DOX: doxorubicin
ncDNA: nuclear DNA
PDT: photodynamic therapy
PTT: photothermal therapy
CDT: chemodymic therapy
PTX: paclitaxel
O_2_^−•^: superoxide anion
